# The Lateral Hypothalamus: An Uncharted Territory for Processing Peripheral Neurogenic Inflammation

**DOI:** 10.3389/fnins.2020.00101

**Published:** 2020-02-12

**Authors:** Marc Fakhoury, Israa Salman, Wassim Najjar, George Merhej, Nada Lawand

**Affiliations:** ^1^Department of Anatomy, Cell Biology and Physiological Sciences, Faculty of Medicine, American University of Beirut, Beirut, Lebanon; ^2^Department of Neurology, Faculty of Medicine, American University of Beirut, Beirut, Lebanon

**Keywords:** hypocretin, lateral hypothalamus, inflammation, orexin, pain

## Abstract

The roles of the hypothalamus and particularly the lateral hypothalamus (LH) in the regulation of inflammation and pain have been widely studied. The LH consists of a parasympathetic area that has connections with all the major parts of the brain. It controls the autonomic nervous system (ANS), regulates feeding behavior and wakeful cycles, and is a part of the reward system. In addition, it contains different types of neurons, most importantly the orexin neurons. These neurons, though few in number, perform critical functions such as inhibiting pain transmission and interfering with the reward system, feeding behavior and the hypothalamic pituitary axis (HPA). Recent evidence has identified a new role for orexin neurons in the modulation of pain transmission associated with several inflammatory diseases, including rheumatoid arthritis and ulcerative colitis. Here, we review recent findings on the various physiological functions of the LH with special emphasis on the orexin/receptor system and its role in mediating inflammatory pain.

## Introduction

The functions of the lateral hypothalamus (LH) in the regulation of vital body functions have become a popular research topic. Important among these functions is that of the regulation of inflammatory pain. The LH is also a site for integration of autonomic and endocrine responses, and a crucial regulator of pituitary function and homeostatic balance (Ferguson and Samson, 2003; Timper and Bruning, 2017). It is divided into three rostro-caudal zones – the anterior LH (aLH), the tuberal LH (tLH), and the posterior LH (pLH) (Berthoud and Munzberg, 2011). The aLH is continuous rostrally with the lateral preoptic area, and extends caudally to the level of the rostral pole of the venteromedial nucleus (VMN). While the tLH is coextensive with the VMN, the pLH follows the tuberal division at the level of the mamillary complex (Bernardis and Bellinger, 1993).

The LH plays a key role in regulating autonomic functions and relays information to all major parts of the brain including the major hypothalamic nuclei (Seoane-Collazo et al., 2015; Stuber and Wise, 2016). Converging evidence from functional, structural, and behavioral studies confirmed the importance of this region not only in regulating metabolism and feeding behavior, but also in serving as a motivation-cognition interface (Stuber and Wise, 2016; Petrovich, 2018). Interestingly, neurons in the LH are the largest in the hypothalamus and are topographically well organized (Bernardis and Bellinger, 1993). Chief among them are the orexin neurons that project widely to the neuraxis and undertake many important functions. A growing body of evidence suggests that orexin neurons play a key role in regulating wakefulness (Eggermann et al., 2003), sleep (Inutsuka and Yamanaka, 2013), food intake (Barson and Leibowitz, 2017), autonomic and endocrine functions (Grimaldi et al., 2014), reward-related behaviors (Espana, 2012) and pain-related behaviors (Inutsuka et al., 2016). However, despite significant progress in research, a more refined understanding of the detailed functions of orexin neurons of the LH in inflammatory pain is needed. This review provides a closer look on the functions and anatomy of LH neurons with an emphasis on the role of the orexin system in pain transmission and inflammatory disorders, and discusses avenues for future research.

## General Functions of the Lateral Hypothalamus

The LH is involved in numerous functions, including sleep, arousal, and the regulation of the autonomic nervous system (ANS) (Gerashchenko and Shiromani, 2004; Szymusiak and McGinty, 2008; Seoane-Collazo et al., 2015). More importantly, it is considered to be a key regulatory center in feeding, hence its description as a “feeding center” (Petrovich, 2018).

LH neurons control feeding, blood pressure, heart rate, water intake and sodium excretion largely through the activation of adrenergic receptors (Shiraishi, 1991; Saad et al., 2000; Mendonca et al., 2018). In addition, they receive inhibitory noradrenergic input from the locus coeruleus, which helps prevent excessive activity in the arousal pathway during the waking cycle (Breton-Provencher and Sur, 2019). Also, beta (β)-adrenoceptors activation by noradrenaline in the LH appears to be involved in the suppression of feeding behavior (Leibowitz, 1970). On the other hand, activation of alpha 1 (α_1_)-adrenoceptors of the LH has been linked to behavioral activation and exploration, despite the insignificant number of these receptors in the LH (Lin et al., 2008).

The LH also plays an important role in the brain reward system. This was demonstrated by studies using intracranial self-stimulation in rodents showing that animals will willingly perform an operant response to receive rewarding pulses of electrical stimulation within the LH (Fakhoury et al., 2016; Ide et al., 2017). The rewarding effect of LH self-simulation is largely influenced by the dopamine and opioid systems as alterations in these systems were shown to either suppress or disrupt the self-stimulation behavior (Koob et al., 1978; Ide et al., 2017). The LH, through GABA neurons, also plays an important role in learning to respond to cues that predict the delivery of a reward (Sharpe et al., 2017). In addition, GABA neurons of the LH highly project to the ventral tegmental area (VTA) (Sharpe et al., 2017), a center rich in dopamine neurons that is known to be crucial for learning, reward processes and feeding behavior (Hommel et al., 2006; Ranaldi, 2014).

Recently, many reports have implicated the LH in the regulation of inflammatory pain (Holden and Pizzi, 2008; Jeong and Holden, 2009a; Wardach et al., 2016). For instance, studies have shown that stimulation of the LH produces analgesic and anti-nociceptive effects in an animal model of inflammatory pain (Jeong and Holden, 2009a), and that this effect is largely due to the activation of α-adrenoceptors in the dorsal horn of the spinal cord (Aimone and Gebhart, 1987; Jeong and Holden, 2009a), and to the involvement of lateral hypothalamic orexin neurons (Inutsuka et al., 2016). The specific role of the LH and orexin neurons in the regulation of pain and inflammation is discussed in details in subsequent sections.

## Lateral Hypothalamic Neuronal Populations

### GABA Neurons

**FIGURE 1:**
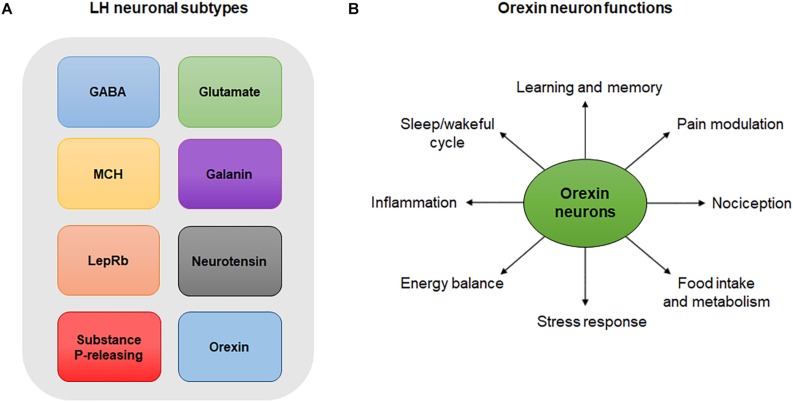
**(A)** LH neuronal subtypes. Simplistic diagram showing existing neuronal populations in the LH. Neuronal populations in the LH include, but are not limited to, GABA neurons, glutamate neurons, MCH-expressing neurons, galanin-expressing neurons, LepRb-expressing neurons, neurotensin-releasing neurons, substance P-releasing neurons and orexin neurons. The degree to which these neuronal populations overlap is not represented in this diagram. **(B)** Orexin neuron functions. Orexin neurons are involved in numerous physiological and behavioral processes including sleep/wakeful cycles, learning, memory, pain, nociception, food intake, metabolism, stress, energy balance and inflammation.

Lateral hypothalamus neurons are composed of many overlapping neuronal populations that play distinct functions in the central nervous system (CNS) (Figure 1A). Studies over the past few decades have largely focused on the functions of GABAergic neurons of the LH and their projections in reward and feeding behavior (Suyama and Yada, 2018). Evidence suggests that these neurons encode information necessary for associating specific cues with reward delivery. In experiments employing optogenetics, a highly specific technique that involves the use of light to activate or inhibit neurons, inhibition of LH GABA neurons was shown to reduce responding to a cue predicting a food reward, indicating that these neurons encode information pertaining to reward prediction (Sharpe et al., 2017). On the other hand, optogenetic inhibition of LH GABA neurons that project to the VTA increased responding to the food reward-paired cue, suggesting that these neurons may play a role in relaying reward-predictive information to other neuronal structures (Sharpe et al., 2017). Interestingly, a study evaluating the role of LH GABA neurons projecting to the VTA showed that optogenetic activation of this pathway can either induce a feeding or rewarding effect depending on the frequency of the stimulation used (Barbano et al., 2016). Last but not least, a recent study by Giardino et al. (2018) found that the bed nucleus of the stria terminalis sends two non-overlapping GABAergic projections to the LH that express several neuropeptides including corticotropin-releasing factor (CRF) and cholecystokinin.

### Glutamate Neurons

Glutamate neurons of the LH mediate important physiological processes in the CNS. Studies have shown that LH glutamate neurons produce behavioral functions opposite to those of LH GABA neurons. In mice, optogenetic activation of putative glutamate neurons of the LH suppressed feeding and produced aversion-related phenotypes (Jennings et al., 2013), while the opposite effect was observed following optogenetic or chemogenetic activation of LH GABA neurons (Jennings et al., 2015). The opposite functions of LH glutamate and GABA neurons in feeding and reward-related processes are mainly explained by differences in their projection pattern. Indeed, glutamate neurons of the LH send dense projections to the lateral habenula (LHb), a region involved in processing aversive stimuli (Stamatakis et al., 2016), in contrast to LH GABA neurons, whose projections mainly target the VTA (Barbano et al., 2016). Besides its role in the regulation of feeding and aversion-related behaviors, LH glutamate neurons have been implicated in compulsive (Mangieri et al., 2018) and hyperkinetic (Schneeberger et al., 2018) behaviors.

### Melanin-Concentrating Hormone (MCH)-Expressing Neurons

Neurons expressing melanin-concentrating hormone (MCH) are also widely present in the LH. Functional and anatomical studies showed that MCH neurons co-express the vesicular glutamate transporter 2 (VGLUT2), indicating a glutamatergic identity (Schneeberger et al., 2018), and project to several regions of the CNS ranging from the cortex to the spinal cord (Bittencourt and Elias, 1998). MCH is an orexigenic hypothalamic peptide that exerts inhibitory effects on lateral hypothalamic neurons (Gao, 2009). Although the mechanism of action of MCH is yet to be fully determined, its inhibitory effect on LH neurons is largely mediated by the attenuation of excitatory glutamate transmission presynaptically (Gao, 2009). MCH was also shown to depress synaptic activity of LH GABA neurons, suggesting a substantial level of complexity in its modulation of LH neuron activity (Gao and van den Pol, 2001). Mounting evidence suggests that MCH neurons directly regulate feeding behavior. Indeed, both administration of MCH (Qu et al., 1996; Clegg et al., 2003) and activation of MCH receptors (Shearman et al., 2003) increases food intake and facilitates body weight gain in rodents. Conversely, genetic knockout of the MCH gene (Shimada et al., 1998) or pharmacological blockade of MCH receptors (Shearman et al., 2003) leads to substantial decreases in food intake in mice. In addition, through their direct projections to gonadotropin-releasing hormone (GnRH) synthesizing neurons, MCH neurons convey critical homeostatic signals to the reproductive axis, and contribute considerably to the functional connection between the regulation of food intake and reproduction (Gao and van den Pol, 2001; Skrapits et al., 2015).

### Galanin and Leptin-Receptor Expressing Neurons

Another type of neuron found in the LH is the galanin-containing neuron (Qualls-Creekmore et al., 2017). These neurons represent a GABAergic subpopulation of LH neurons with a distinct molecular phenotype and projection pattern. Unlike GABAergic neurons of the LH, which project to the VTA, galanin neurons of the LH lack direct VTA innervation (Qualls-Creekmore et al., 2017). Instead, galanin neurons of the LH strongly innervate the locus coeruleus (Laque et al., 2015), a site involved in the control of arousal (Gonzalez and Aston-Jones, 2006; Sara and Bouret, 2012) and reward processing (Bouret and Richmond, 2015; Hofmeister and Sterpenich, 2015). Galanin is a 29 amino acid neuropeptide widely distributed in the brain (Skofitsch and Jacobowitz, 1986; Gentleman et al., 1989; Perez et al., 2001) that acts as an inhibitor of synaptic transmission in the hypothalamus (Kinney et al., 1998). Galanin also acts in the hypothalamus to produce behavioral hyperalgesia through activation of two descending pronociceptive pathways; one that involves the medullary dorsal reticular nucleus, and another one that involves serotonin neurons acting on the spinal cord (Amorim et al., 2015). Galanin has also been reported to promote feeding behavior. Indeed, chemogenetic activation of LH galanin neurons (Qualls-Creekmore et al., 2017) or central injection of galanin (Kyrkouli et al., 1990) enhances food-seeking behavior, while targeted knockout of the galanin gene (Adams et al., 2008) or the galanin receptor (Zorrilla et al., 2007) reduces dietary fat intake.

Another neuronal population of the LH involved in the regulation of feeding behaviors is the leptin-receptor (LepRb) expressing neuron. LepRb-expressing neurons are widely expressed in the brain, but are particular enriched within the hypothalamus and the brainstem (Elmquist et al., 1998). In mice, leptin acts on LepRb-expressing neurons of the LH to decrease feeding and body weight (Leinninger et al., 2009). LepRb-expressing neurons of the LH also innervate the VTA, and leptin action on these neurons increases VTA dopamine neuron activity, suggesting a link between the anorexic effect of leptin and the mesolimbic DA system (Leinninger et al., 2009). Interestingly, LepRb-expressing neurons in the LH are thought to be GABAergic (Leinninger et al., 2009), and a subpopulation of these neurons in the LH was shown to co-express the inhibitory acting neuropeptide galanin (Laque et al., 2013), suggesting that the anorexic effect of leptin is likely due to its interaction with other neuropeptidergic receptors in the LH.

### Substance P and Neurotensin-Releasing Neurons

Substance P and neurotensin-releasing neurons are also found in the LH (Ljungdahl et al., 1978; Yamano et al., 1986). Substance P is a member of the tachykinin neuropeptide family and is associated with multiple physiological processes including wound healing, neurogenic inflammation and tissue homeostasis (Suvas, 2017). In the LH, substance P-containing neurons have been proposed to exert antinociceptive functions by activating spinally projecting serotonin neurons in the rostral ventromedial medulla (RVM) (Holden and Pizzi, 2008). These cells activate spinally projecting serotonin neurons either through direct contact, or indirectly through the innervation of interneurons in the RVM, thereby altering nociceptive responses in the dorsal horn of the spinal cord (Holden and Pizzi, 2008).

On the other hand, neurotensin neurons of the LH are involved in the regulation of the sleep/wake cycle (Gerashchenko and Shiromani, 2004) and are implicated in feeding behavior (Woodworth et al., 2017) and reward processes (Kempadoo et al., 2013). Studies exploring the role of neurotensin in reward and feeding have indicated that this neuropeptide promotes reward by enhancing glutamate transmission in the mesolimbic dopaminergic system (Kempadoo et al., 2013) and promotes weight loss by suppressing the increased appetitive drive through activation of the G-protein-coupled neurotensin receptor-1 (Woodworth et al., 2017). Finally, neurotensin neurons of the LH have been implicated in a number of other physiological processes, including hyperthermia and energy balance, though the central mechanisms by which these processes are mediated remain to be fully elucidated (Brown et al., 2018; Naganuma et al., 2019).

### Orexin Neurons and Other Neuronal Populations of the LH

By being an extensively researched population of cells in the past recent years, orexinergic neurons, and especially those of the LH, have been shown to have different roles in inflammatory pain and in the balance of psychological functions (Figure 1B). Orexinergic neurons synthesize two neuropeptides (Orexin A and B) from the precursor prepro-orexin (Chieffi et al., 2017). Furthermore, inhibition of orexin neurons by local GABAergic neurons of the LH is thought to disrupt the sleep cycle (Ferrari et al., 2018). On the other hand, inhibition of orexin neurons through activation of acetylcholine and dynorphin promotes wakefulness (Ferrari et al., 2018). The anatomy and distribution of orexin neurons and peptides as well as their functions in pain and inflammation is discussed in details in later sections.

Other neuronal populations in the LH not mentioned above include neurons that express cocaine- and amphetamine-regulated transcript, thyrotropin-releasing hormone, encephalin, urocortin-3 and corticotropin-releasing hormone (for review see Bonnavion et al., 2016; Tyree and de Lecea, 2017).

## The LH and the Hypothalamus-Pituitary-Adrenal Axis (HPA) in the Regulation of Inflammation and Stress Response

Inflammation is a biological response of the immune system that is triggered when the tissue is altered by any form of injury. It is characterized by the release of cytokines and by a noticeable change in the number of white blood cells (Zhang and An, 2007; Stiegel et al., 2016). Cytokines relay important inflammatory signals that initiate and maintain the inflammatory response along the cell signaling pathway (Zhang and An, 2007). This inflammatory response can ultimately alter the corresponding body organ function and induce a number of inflammatory-associated diseases (Chen L. et al., 2018; Jeon and Kim, 2018). Numerous studies indicate that the LH plays a significant role in the regulation of inflammatory pain. In particular, findings suggest that stimulation of the LH produces analgesic and anti-nociceptive effects in models of inflammatory pain (Jeong and Holden, 2009a; Jahangirvand et al., 2016; Wardach et al., 2016). Consistently, lateral hypothalamic nuclei were shown to receive dense nociceptive inputs from the spinothalamic tract, which conveys information related to pain (Dado et al., 1994).

In addition to its role in the regulation of inflammatory pain, the LH has been shown to regulate stress response though its functional connection with the HPA axis (Bonnavion et al., 2015; Mokhtarpour et al., 2016). The HPA axis is a complex set of neuronal connections that relay the hypothalamus to the pituitary gland, and the pituitary gland to the adrenal gland above the kidney (Herman et al., 2016). It mainly responds bi-directionally, either stimulating or inhibiting the secretion of certain hormones. This system is mainly activated when subject to a stressful event. It also maintains body balance at several levels, including metabolic, immune and endocrine. Any alteration to the HPA axis leads to stress response alterations (Kyrou and Tsigos, 2009). Hypophysiotropic neurons, which are found in the paraventricular nucleus (PVN), synthesize and secrete CRF. Stress responses stimulate the release of CRF into the hypophysial portal vessels, which in turn stimulate the anterior pituitary gland to release adrenocorticotropic hormone (ACTH). This hormone then acts on the adrenal cortex, its main target, which will stimulate the zona fasciculata to synthesize and secrete glucocorticoids (Gallo-Payet et al., 2017). Glucocorticoids are responsible for regulating physiological changes in the body and activating the HPA axis through intracellular receptors (Smith and Vale, 2006).

Numerous studies indicate that the HPA axis is highly influenced by inflammation and stress responses (Stephens and Wand, 2012; Chen et al., 2017). Activation of the HPA axis occurs through the corticotropin releasing hormone (CRH), which binds to CRH1 and CRH2 receptors, whose activation regulate several behavioral and physiological processes including anxiety and sleep (Reul and Holsboer, 2002). Activation of CRH receptors also lead to the excitation of orexin neurons (Winsky-Sommerer et al., 2004). These orexin neurons interfere with the arousal stress response states, and play a role in the modulation of the HPA axis (Messina et al., 2016). In particular, they mediate stress responses by regulating the release of CRH and by mediating the release of corticosterone and ACTH (Mokhtarpour et al., 2016; Grafe et al., 2017). On the other hand, the excitation of orexin and the release of corticosterone in the LH during stress responses is suppressed by leptin, a satiety hormone which acts through a network of leptin-sensitive GABA neurons within the LH (Bonnavion et al., 2015). Orexin, leptin and GABA neurons of the LH all play a crucial role in balancing the HPA axis, either directly or indirectly by acting on intermediate structures (Bonnavion et al., 2015).

Altogether, the findings mentioned above indicate that the LH, in conjunction with the HPA axis, functions to coordinate inflammation and stress responses in the CNS. In the following sections, a particular emphasis will be given to the anatomy and distribution of LH orexin neurons and their role in pain and inflammation.

## Orexin Neurons: Structure, Transmitters and Co-Localization

Orexin, also known as hypocretin, is a neuropeptide secreted by orexin neurons in the lateral hypothalamic area. They are two types of orexin; orexin A (also referred to as hypocretin-1) and orexin B (also referred to as hypocretin-2). These neuropeptides originate from the same precursor known as prepro-orexin (Chieffi et al., 2017). Orexin-A is a 33-amino-acid peptide while orexin-B is a 28-amino-acid peptide (Chieffi et al., 2017). Both orexin A and orexin B bind to the G-protein coupled receptors orexin receptor 1 (OX-1) and 2 (OX-2) (also known as hypocretin receptors type 1 and 2) (Smart and Jerman, 2002). Orexin A binds to both OX-1 and OX-2 with the same affinity while orexin B has a higher affinity for OX-2 over OX-1 receptors (Sakurai et al., 1998).

In the CNS, orexin is co-localized with other transmitters, some of which include dynorphin (Chou et al., 2001), glutamate (Abrahamson et al., 2001; Torrealba et al., 2003), galanin (Barson et al., 2011) and prolactin (Risold et al., 1999). Experiments employing *in situ* hybridization and immunohistochemical techniques indicate that orexin neurons in the LH mostly express the vesicular glutamate transporters, VGLUT1 and VGLUT2, suggesting that they are glutamatergic (Rosin et al., 2003).

Orexin neurons project their axons to most parts in the brain and spinal cord, especially to areas that are involved in the modulation of pain (Watanabe et al., 2005). In addition, orexin neurons in the LH send projections to multiple sites related to arousal including the serotonergic dorsal raphe (Brown et al., 2002). Orexin neurons also project to the tuberomammillary nucleus (TMN) (Torrealba et al., 2003), a center involved in the control of arousal, learning and memory (Huston et al., 1997; Sakai et al., 2010). Pre-synaptically, orexin increases the release of glutamate and GABA in the hypothalamus, while post-synaptically, it increases Ca^2+^ levels, thus leading to the depolarization, hence activation, of TMN neurons by glutamatergic orexin terminals (Torrealba et al., 2003).

Last but not least, orexin neurons have been shown to directly interact with neuropeptide Y (NPY), a peptide that plays a role in the regulation of feeding behavior, metabolism and energy balance (Beck, 2006). This neuropeptide is primarily synthesized by neurons in the arcuate nucleus (ARC) and is present in different areas of the brain including the cortex, hippocampus, hindbrain and hypothalamus (Beck, 2006). Through its heavy projections to the ARC (Guan et al., 2001), orexin neurons interact with NPY to regulate numerous physiological processes and behaviors including food intake and Ca^2+^ signaling (Yamanaka et al., 2000; Muroya et al., 2004).

## Orexin Receptors and Neuropeptide Distribution

The distribution of OX-1 and OX-2 receptors has been established in different species including rats and mice. Studies employing *in situ* hybridization, immunohistochemistry and quantitative reverse transcription–polymerase chain reaction in rodents found that these receptors are widely distributed throughout the brain and spinal cord (Trivedi et al., 1998; Hervieu et al., 2001). Although some overlap exist in the distribution pattern of OX-1 and OX-2 receptors, these receptors are differentially expressed in the CNS (Trivedi et al., 1998; Marcus et al., 2001).

OX-1 receptors are primarily expressed in the ventromedial hypothalamic nucleus, prefrontal and infralimbic cortex, hippocampus, paraventricular thalamic nucleus, dorsal raphe, and locus coeruleus (Trivedi et al., 1998; Hervieu et al., 2001; Marcus et al., 2001), and to a lesser extent in the medial preoptic area, lateroanterior and dorsomedial hypothalamic nuclei, lateral mammillary nucleus and posterior hypothalamic area (Trivedi et al., 1998). They are also found in the periaqueductal gray and dorsal root ganglia, which suggests a role in the regulation of pain (Hervieu et al., 2001; Ho et al., 2011), and in the spinal cord, which suggests a role in the regulation of the parasympathetic and sympathetic system (Hervieu et al., 2001). On the other hand, OX-2 receptors are predominantly expressed in the TMN, PVN, cerebral cortex, NAc, subthalamic and paraventricular thalamic nuclei, septal nuclei, raphe nuclei, and anterior pretectal nucleus, and to a lesser extent in the ventromedial/dorsomedial hypothalamic nuclei and the posterior and lateral hypothalamic areas (Trivedi et al., 1998; Marcus et al., 2001).

Interestingly, a study examining the expression of OX-1 and OX-2 receptors mRNA with *in situ* hybridization in rats and mice found some species-specific differences (Ikeno and Yan, 2018). For instance, OX-1 receptors are expressed in the caudate putamen and ventral TMN in rats only, while they are detected in the bed nucleus of the stria terminalis, medial division, posteromedial part in mice only (Ikeno and Yan, 2018). On the other hand, OX-2 receptors show similar pattern of expression between the two species, though they are more widely expressed in the ventral TMN of rats compared to mice (Ikeno and Yan, 2018). This differential distribution of orexin receptors is consistent with the proposed multifaceted roles of orexin in regulating homeostasis and other functions in the CNS.

Orexin A and B neuropeptides, as demonstrated in the literature, are also widely expressed in different regions of the brain and spinal cord. Findings from immunohistochemical and radioimmunoassay techniques indicate that orexin A fibers are found throughout the hypothalamus, septum, thalamus, locus coeruleus and spinal cord, and in the paraventricular and supraoptic nucleus (Cutler et al., 1999; Date et al., 2000; Bingham et al., 2001; Nixon and Smale, 2007). In addition, orexin A fibers colocalize with substance P positive afferents of dorsal root ganglia neurons, which further strengthens its confirmed role in the regulation of pain (Colas et al., 2014). On the other hand, orexin B fibers are distributed sparsely in the hypothalamus and the spinal cord (Cutler et al., 1999; Date et al., 2000), but are absent in the paraventricular and supraoptic nucleus (Nixon and Smale, 2007). Interestingly, a study investigating the distribution of orexin A and orexin B in the brain of nocturnal and diurnal rodents found striking differences among species, in particular in the lateral mammillary nucleus, ventromedial hypothalamic nucleus and flocculus (Nixon and Smale, 2007).

## Role of Orexin Neurons in Pain Modulation

Orexin neurons play multifaceted functions in the CNS. Not only do they play a role in the regulation of sleep/wakeful cycle (Inutsuka and Yamanaka, 2013), feeding behavior (Barson and Leibowitz, 2017), endocrine system (Taylor and Samson, 2003), stress response (Sargin, 2019), energy homeostasis (Sakurai, 2003) and rewarding behavior (Espana, 2012), they also play a role in the regulation of cognitive functions including learning and memory (Telegdy and Adamik, 2002; Akbari et al., 2007; Sharf et al., 2010; Aitta-Aho et al., 2016; Mavanji et al., 2017). More importantly, orexin neurons have been shown to play a crucial role in the modulation of pain transmission (Razavi and Hosseinzadeh, 2017).

First of all, orexin neurons project to areas involved in pain and sensation. Indeed, studies employing *in situ* hybridization and immunohistochemical techniques have shown that orexin receptors are present in high amounts in the NAc, dorsal root ganglia and spinal cord (Trivedi et al., 1998; Hervieu et al., 2001). All of these areas are known to modulate pain response (Thomas Cheng, 2010; Chang et al., 2014; Guha and Shamji, 2016), suggesting that orexin neurons are highly involved in pain regulation and nociceptive perception. The presence of heavy projections from orexin neurons of the lateral hypothalamic area to the dorsal horn of the spinal cord strongly implicates orexin neurons in nociceptive processing (van den Pol, 1999). In addition, localization of the fibers of orexin-containing neurons in the hypothalamus, locus coeruleus, thalamus and periaqueductal gray is consistent with their crucial roles in sensory processing (Marcus and Elmquist, 2006).

In animal models of inflammatory pain, administration of orexin receptor antagonists in the NAc or VTA was shown to decrease the LH-induced antinociceptive effect, indicating a role of orexin neurons projecting to the mesolimbic system in the modulation of inflammatory pain (Sadeghi et al., 2013; Ezzatpanah et al., 2016; Jahangirvand et al., 2016). Recent studies have also highlighted the dual integrative role of orexin neurons in nociceptive perception and analgesic regulation (Inutsuka et al., 2016). Evidently, orexin modulates pain perception at both spinal and supra-spinal levels (Razavi and Hosseinzadeh, 2017). Indeed, experiments in rodents showed that orexin-A neurons that project to the dorsal horn of the spinal cord mediates the antinociceptive effect of posterior hypothalamic stimulation (Jeong and Holden, 2009b), and that activation of orexin-1 receptor in the spinal cord suppresses pain responses in rodents (Yamamoto et al., 2002, 2003b; Kajiyama et al., 2005). At the supraspinal level, orexin was shown to act on several sites, including the periaqueductal gray (PAG) (Chen Y.H. et al., 2018) and the RVM (Azhdari-Zarmehri et al., 2014), to modulate pain perception.

In support of the notion that the orexin system plays a role in pain modulation, intrathecal administration of orexin A or B (Azhdari Zarmehri et al., 2011; Ghasemi et al., 2015; Azhdari-Zarmehri et al., 2018) and pharmacogenetic activation of orexin neurons (Inutsuka et al., 2016) was shown to produce anti-nociceptive and analgesic effects in experimental animals, respectively. Evidence also indicates that intrathecal or intracerebroventricular injection of orexin-A produces anti-mechanical allodynic effect in a rat model of neuropathic pain (Yamamoto et al., 2003a). It is important to note, however, that the anti-nociceptive effects of orexin A are more remarkable than those of orexin B, indicating that orexin-1 receptors are more involved than orexin-2 receptors in the regulation of nociceptive transmission (Yamamoto et al., 2002; Mobarakeh et al., 2005). In addition, the anti-nociceptive effects of orexin A were shown to be suppressed by administration of either dopamine (Okumura et al., 2015) or adenosine (Mobarakeh et al., 2005; Okumura et al., 2016) receptor antagonists, suggesting a possible involvement of the adenosine and dopamine pathways in orexin-induced antinociceptive actions. Accordingly, specific ablation of orexin neurons in mice was shown to increase pain perception generated by mechanical, thermal and chemical noxious stimuli (Inutsuka et al., 2016). Conversely, pharmacogenetic activation of orexin neurons was shown to induce analgesia in experimental animals (Inutsuka et al., 2016). Studies have also demonstrated the involvement of the endocannabinoid signaling in the analgesic and antinociceptive effects of orexin neurons (Ho et al., 2011; Lee et al., 2016).

Overall, stimulation of orexin neurons in the hypothalamus by peripheral inflammation and stressful conditions produces analgesic effects by activating descending inhibitory pathways. This analgesic effect is largely mediated by descending pathways from the hypothalamus to the dorsal horn of the spinal cord through the release of oxytocin, a neuropeptide elaborated by the hypothalamic paraventricular and supraoptic nuclei (Eliava et al., 2016). Release of oxytocin onto sensory spinal cord neurons in an animal model of inflammatory pain was previously shown to suppress nociception and promote analgesia (Eliava et al., 2016). One important supraspinal site of orexin pain modulation is the PAG (Razavi and Hosseinzadeh, 2017). Also, orexin neurons of the hypothalamus control nociceptive processing by receiving inputs from the dorsal horn of the spinal cord and from the adenosine and dopamine pathways, and through its interaction with the endocannabinoid system.

## Role of the Orexin System in Inflammatory Disorders

Owing to its important role in the regulation of inflammatory pain, it is not surprising that the orexin system has been implicated in the underlying mechanisms of a number of inflammatory disorders including rheumatoid arthritis and ulcerative colitis. Rheumatoid arthritis is a chronic inflammatory disease that primarily affects the lining of the synovial joints, with symptoms ranging from pain and stiffness to muscle weakness and weight loss (Combe, 2009). In a rat model of rheumatoid arthritis, intravenous administration of orexin A was shown to reduce pain sensation as well as the serum level of nerve growth factor (NGF), a major mediator of inflammatory and neuropathic pain (Mohamed and El-Hadidy, 2014); effects that are likely attributed to the activation of OX-1 receptors (Yamamoto and Shono, 2007). Accordingly, the expression of OX-1 receptors was found to be decreased in rheumatoid arthritis, indicating that these receptors may constitute a critical target for the treatment of this disease (Sun et al., 2018). Besides its role in arthritis, the orexin system was shown to be involved in the pathophysiology of ulcerative colitis. Ulcerative colitis is a chronic type of inflammatory bowel disease that causes damage in the mucosa and the superficial submucosa of the colon and the rectum resulting in inflammation of the large intestine (Fakhoury et al., 2014). Immunostaining of OX-1 receptors in human colonic mucosa show that OX-1 receptors are present in the inflamed mucosa of patients with ulcerative colitis, but absent in normal colon (Messal et al., 2018). In addition, intraperitoneal administration of orexin A in mouse models of colitis was shown to result in significant reductions in symptoms of ulcerative colitis, including colon length reduction and weight loss, and led to a marked reduction in the level of pro-inflammatory cytokines including IFN-gamma, IL-6, and TNF alpha (Messal et al., 2018). Thus, OX-1 receptors, along with orexin A, induce an anti-inflammatory effect by inhibiting the production of pro-inflammatory cytokines, thereby protecting the epithelium from inflammatory damage (Messal et al., 2018).

In addition, the orexin system has been involved in a number of other diseases where inflammation constitutes a key pathological feature; these include Multiple sclerosis (MS), Huntington’s disease (HD), Parkinson’s disease (PD), and Alzheimer’s disease (AD). In particular, findings indicate that patients with MS have significantly lower serum (Gencer et al., 2019) and cerebrospinal fluid (CSF) (Kato et al., 2003) levels of orexin A compared to normal values, suggesting that the orexin system could be an interesting target for MS treatment. Consistently, peripheral administration of orexin A in mice undergoing experimental autoimmune encephalomyelitis (EAE), a well-established model of MS, was shown to induce anti-inflammatory and neuroprotective effects, suggesting that it might constitute a potential therapeutic approach in MS (Becquet et al., 2019). On the other hand, in R6/2 mice, a well-established mouse model of HD, Petersen et al. (2005) reported a dramatic atrophy of orexin neurons in the LH and a significant decrease in the CSF level of orexin A (Petersen et al., 2005). A decrease in orexin-immunopositive neurons was also found in the hypothalamus of five HD patients (Petersen et al., 2005). Paradoxically, a more recent clinical report found no differences between the CSF levels of orexin A in patients with HD compared to healthy individuals (Baumann et al., 2006). Such discrepancy in the results could be attributed to differences in the method employed to quantify changes in the orexin system. In patients with PD, studies showed a significant decrease in the number of orexin neurons (Fronczek et al., 2007) and the level of orexin in ventricular CSF (Drouot et al., 2003). However, these results are inconsistent with reports indicating that CSF orexin level are not disturbed in PD (Ripley et al., 2001; Overeem et al., 2002), suggesting a complex role of the orexin system in the underlying pathophysiology of PD. Finally, studies concerned with the role of the orexin system in AD showed that CSF levels of orexin are elevated in AD patients compared to control individuals (Wennstrom et al., 2012; Liguori et al., 2016, 2017). On the contrary, another report showed that the number of orexin A-immunoreactive neurons and the concentration of orexin A in the CSF is markedly reduced in AD patients (Fronczek et al., 2012), suggesting some degree of complexity in the relationship between the orexin system and AD. Clearly more work is needed to better understand the role of the orexin system in the underlying pathophysiology of AD and other disorders characterized by aberrant inflammatory response.

## Conclusion

The neuronal network of the LH has been implicated in many vital physiological functions in the body such as feeding behavior, reward systems, sleep/wakefulness cycle, stress regulation and inhibition of inflammatory pain. Integrated in this network are orexin neurons; though few in numbers, they play a big part in controlling several aspects of bodily functions because of their extensive projections in the CNS and the abundance of orexin receptors in peripheral organs. The role of these neurons in mediating inflammatory processes and regulating pain perception has been highlighted in several studies, however, more experiments are needed to better understand the signaling mechanisms underlying their anti-inflammatory and anti-nociceptive effects. Future work should also focus on better understanding the role of the orexin system in inflammatory disorders. The multifunctional role of orexin receptors makes them a credible therapeutic target for disorders characterized by inflammatory pain and could ultimately be used in clinical practice to help patients cope with their symptoms.

## Author Contributions

MF and IS contributed to the initial design of the review and wrote the first draft of the manuscript. WN and GM wrote sections of the manuscript. All authors contributed to manuscript revision, read, and approved the submitted version.

## Conflict of Interest

The authors declare that the research was conducted in the absence of any commercial or financial relationships that could be construed as a potential conflict of interest.
